# Latent profile analysis and influencing factors of proactive health behaviors in hypertensive patients from the perspective of the health belief model

**DOI:** 10.3389/fpubh.2026.1789975

**Published:** 2026-02-23

**Authors:** Zheyuan Xia, Yukuan Miao, Leran Tang, Ting Yao, Qiao Hu, Xiao Wang, Xiang Wang

**Affiliations:** 1 The School of Nursing, Anhui University of Chinese Medicine, Hefei, China; 2 Laboratory of Geriatric Nursing and Health, Anhui University of Chinese Medicine, Hefei, China; 3 Emergency Intensive Care Unit (EICU), The First Affiliated Hospital of Anhui Medical University, Hefei, China

**Keywords:** health belief model, hypertension, latent profile analysis, nursing care, proactive health

## Abstract

**Aim:**

To identify latent profiles of proactive health behaviors in patients with hypertension, examine the category-specific influencing factors.

**Background:**

Proactive health behavior, as an emerging concept, refers to a self-motivated approach to systematically managing health-related factors in order to actively maintain and promote one’s health status. However, existing studies have largely focused on describing the overall level of such behaviors among patients with hypertension, with insufficient exploration of behavioral heterogeneity within this population. Moreover, there has been a lack of systematic integration of established behavioral theories to explain the multifactorial mechanisms underlying different behavioral patterns, which limits the development of precise nursing interventions.

**Methods:**

A cross-sectional study was performed, involving 352 patients with hypertension from 8 communities in Anhui Province from September to December 2025. The survey tools included self-designed demographic and clinical instrument, the Proactive Health Behavior Scale for Hypertensive Patients, the Self-Efficacy Scale for Hypertensive Patients, the Health Literacy Management Scale (HeLMS). Latent profile analysis (LPA) was used to identify subtypes of proactive health behavior among hypertension patients. Multinomial logistic regression analysis was applied to determine the factors associated with the identified subtypes.

**Results:**

A total of 352 questionnaires were distributed, yielding 321 valid responses (a response rate of 91.2%). The total score of proactive health behavior was 89.57 ± 22.99 points. The LPA revealed four profiles of proactive health behavior: the positive proactive health behavior profile (Class 1, *n* = 50, 15.8%), the self-regulating proactive health behavior profile (Class 2, *n* = 114, 35.4%), the medically compliant-proactive health behavior profile (Class 3, *n* = 96, 30.2%), and the passive proactive health behavior profile (Class 4, *n* = 61, 18.6%). The entropy value was high (0.856), indicating a correct classification. Multivariate regression analyses showed that age, educational level, marital status, employment status, disease duration, hospitalization due to hypertension, self-management level, self-efficacy level and health literacy as factors influencing proactive health behavior profiles.

**Conclusion:**

The proactive health behavior among hypertension patients was at a moderate level, revealing four distinct behavioral categories with significant differences. Guided by the Health Belief Model, profile-specific influencing factors were analyzed, which informed the development of tailored intervention strategies.

## 1 Introduction

Hypertension is a major global public health challenge. According to the largest global trend analysis report on hypertension to date, the number of individuals with hypertension aged 30–79 years has doubled from 650 million to 1.28 billion over the past three decades (1). Blood pressure control largely depends on patient-led health behaviors, which encompass not only fundamental health maintenance practices such as diet management, physical activity, and emotional regulation but also hypertension-specific behaviors including regular medication adherence and consistent blood pressure monitoring. Studies have demonstrated a significant positive correlation between blood pressure control levels and the extent to which patients implement these health behaviors (2–4). Therefore, strengthening patient-led health behaviors has become a key pathway to improving blood pressure control outcomes.

Although previous studies have achieved certain progress in improving health behaviors such as medication adherence, self-management skills, self-efficacy, and health literacy among patients, existing research still presents notable limitations. First, most studies focus on describing the overall level of patient health behaviors or analyzing independent effects of single influencing factors, without fully revealing the behavioral heterogeneity that may exist across different patient subgroups. Specifically, it remains unclear whether potential patterns of proactive health behaviors exist among patients based on multidimensional characteristics such as demographic factors (e.g., age, education level), clinical factors (e.g., disease duration, hospitalization history), and psychosocial status (e.g., self-efficacy, health literacy), as well as the behavioral distinctions among these potential subgroups. Second, the mechanisms driving health behaviors are multidimensional and complex. Yet, existing studies often lack a robust theoretical foundation, failing to systematically apply established behavioral theories to explain the drivers behind distinct behavioral subtypes. This theoretical gap limits a deeper understanding of what motivates behavioral differences among patients and, consequently, constrains the development of interventions precisely tailored to the characteristics of each subgroup.

As an emerging concept, proactive health behavior emphasizes that individuals should act as the “primary agents” of their own health. This involves taking personal responsibility for health-related matters, integrating accessible health resources, and actively managing risk factors such as diet, exercise, and emotional well-being to maintain and enhance health status (5). For hypertension patients, proactive health behaviors encompass not only basic disease management practices such as lifestyle adjustments, regular medication adherence, and routine blood pressure monitoring but also broader competencies including the development of self-health responsibility awareness and proactive identification of health risks (6). This multidimensional conceptual framework offers a novel perspective for systematically identifying potential behavioral subgroups among different patient populations and analyzing intergroup differences. Such an approach helps address the current research gap related to insufficient attention to behavioral heterogeneity.

The Health Belief Model (HBM), a classic theoretical framework for health behavior, systematically explains individual health-related decisions through its core constructs: perceived susceptibility, severity, benefits, and barriers, along with self-efficacy and cues to action. Its strong applicability in chronic disease management has been well documented (7–9). Introducing the HBM into the study of proactive health behaviors in patients with hypertension can therefore address the prevailing theoretical gap. More importantly, it provides a robust conceptual basis for elucidating the mechanisms that drive behavioral differentiation into distinct subgroups within this population.

To address these gaps, this study adopted a person-centered analytical approach. First, Latent Profile Analysis (LPA) was employed to identify heterogeneous subtypes of proactive health behavior across multiple dimensions in a hypertensive patient sample (10, 11). Second, guided by the HBM, we examined the multifactorial mechanisms underlying the formation of these distinct behavioral profiles. Together, these analyses provide an empirical basis for developing stratified interventions and enhancing the precision of health management in this population.

## 2 Subjects and methods

### 2.1 Study design and participants

This cross-sectional study employed a convenience sampling method to recruit participants from eight communities in Anhui Province, China, between September and December 2025. A total of 352 patients with hypertension were included. Inclusion criteria were: (1) a diagnosis of hypertension as defined by the Chinese Guidelines for Hypertension Prevention and Control (2024 Revision) (12), i.e., systolic blood pressure (SBP) ≥ 140 mmHg and/or diastolic blood pressure (DBP) ≥ 90 mmHg; (2) age ≥ 18 years; (3) a disease duration of at least 6 months; (4) clear consciousness and adequate literacy and communication skills for daily life; (5) voluntary informed consent to participate. Exclusion criteria were: (1) a history of mental illness, severe cognitive impairment, or significant visual, auditory, or language impairments; (2) severe cardiac, hepatic, or renal dysfunction, or other major comorbidities; (3) concurrent participation in other similar intervention studies.

The sample size was calculated using the formula for estimating a single population proportion: *n* = Z^2^*p*(1 − p)/e^2^. Assuming a prevalence of hypertension (P) of 27.5% in adults based on “Chinese Hypertension Prevention and Treatment Guidelines (2024 Revised Edition)” (12), a confidence level of 95% (*α* = 0.05), and a margin of error (d) of 5%, the minimum required sample size was 306. Accounting for an anticipated non-response rate of 15%, the final sample size was inflated to 352 participants.

### 2.2 Study procedure

The study was conducted by a team of seven trained members: two research coordinators, one assistant, two quality controllers, and two data analysts. All members possessed at least 2 years of experience in community epidemiological surveys and underwent standardized training prior to data collection. Training content included questionnaire item interpretation (with emphasis on clarifying ambiguous items), standardized communication techniques (e.g., adapting explanations to participants of different educational levels), and methods for identifying and correcting data entry errors. Competency was assessed via a mock survey consisting of a simulated patient interview (to evaluate adherence to communication protocols) and a data entry exercise requiring 100% accuracy. Only members passing all assessments participated in the formal survey.

All participants provided written informed consent prior to enrollment. For participants with limited literacy (e.g., primary education or below, or visual impairment) who were unable to read the consent form independently, investigators verbally presented the entire document in a clear and neutral manner to ensure full understanding and voluntary agreement. Subsequently, in the presence of an independent witness (a non-team member, such as a family member or community worker), these participants affixed a fingerprint as a signature. Both the witness and the investigator then signed the form to attest that the consent procedure was properly completed.

Surveys were administered either at community health centers or participants’ homes using paper questionnaires distributed and collected on-site. Target completion time was maintained at 15–25 min. After individually explaining the study purpose, procedures, and confidentiality, investigators allowed most participants to complete the questionnaire independently. For those unable to do so due to age, limited education, or other reasons, investigators provided neutral question-and-answer assistance without leading responses. All questionnaires were collected immediately upon completion.

Within 24 h of collection, two researchers independently reviewed and entered the data. Any unclear responses were clarified with participants promptly. Questionnaires were excluded if the item response rate was below 90%, completion time was under 10 min, or patterned responding was evident. All retained data were double-entered independently by two staff members for subsequent statistical analysis.

### 2.3 Variables and instruments

During the screening and inclusion of influencing factors, this study adopted the proactive health concept as the content basis, extracting relevant factors through computerized literature retrieval. By searching relevant literature, the influencing factors were extracted. Both Chinese and English databases were searched, including CNKI, Wanfang Data, VIP, China Biology Medicine disk (CBM), PubMed, Web of Science, Cochrane Library, Embase, and CINAHL. The search period spanned from the inception of each database to April 2025. Taking PubMed as an example, the search strategy was as follows: (“Hypertension”[Mesh] OR “high blood pressure”[tiab]) AND((“Health Behavior”[Mesh] OR “Health Promotion”[Mesh] OR “Self Care”[Mesh] OR “Patient Compliance”[Mesh]) OR(“proactive health behavior”[tiab] OR “health promoting behavior”[tiab] OR “self-management”[tiab] OR “medication adherence”[tiab] OR “lifestyle modification”[tiab] OR “positive health management”[tiab]) OR(“Self Efficacy”[Mesh] OR “self-efficac*”[tiab] OR “perceived competence”[tiab]) OR(“Health Literacy”[Mesh] OR “health knowledge”[tiab] OR “patient education”[tiab])). Through the literature review, representative factors for each dimension were preliminarily identified. After team discussions, 16 potential factors influencing proactive health behavior in hypertensive patients were ultimately included. Among these, 12 personal trait factors were compiled into a general information questionnaire, while proactive health behavior, self-management, self-efficacy, and health literacy were assessed using established and validated scales.

#### 2.3.1 Demographic and clinical instrument

The epidemiological and clinical assessment instrument was developed based on a review of relevant literature and expert consultation. It collected data on two main categories of variables. Sociodemographic characteristics included sex, age (categorized as 18–44, 45–59, and ≥60 years), education level (high school or below, college or above), marital status (married / single or others), and current employment status (currently employed: yes/no). Clinical characteristics encompassed smoking history (yes/no), drinking history (yes/no), body mass index (BMI), duration and grade of hypertension, history of hypertension-related hospitalization (yes/no), and the presence of hypertension-related comorbidities (yes/no).

#### 2.3.2 Proactive health behavior scale for hypertensive patients

Proactive health behavior was assessed using a scale originally developed by WEI Yi-lin to measure patient initiative in blood pressure control (6). This 30-item instrument comprises five dimensions: self-health responsibility (10 items), dietary management (8 items), physical activity management (3 items), labor and emotional management (7 items), and disease management (2 items). Responses are scored on a 5-point Likert scale ranging from 1 (“not at all”) to 5 (“extremely”), yielding a total score between 30 and 150, where higher scores indicate greater proactive health behavior competence. In the present study, the scale demonstrated good internal consistency, with a Cronbach’s *α* coefficient of 0.833. This aligns with previously reported reliability, where the full scale achieved a Cronbach’s α of 0.948 and subscales ranged from 0.583 to 0.855 (6).

#### 2.3.3 Self-management behavior scale for hypertensive patients

Blood pressure management behavior was measured using a scale developed by ZHAO Qiu-li to assess patients’ daily self-management practices (13). The scale consists of 33 items across six dimensions: medication management (4 items), blood pressure monitoring (4 items), diet management (10 items), exercise management (3 items), work-rest management (5 items), and emotional management (7 items). Items are rated on a 5-point Likert scale from 1 (“not at all”) to 5 (“extremely”), with total scores ranging from 33 to 165. Higher scores reflect better hypertension self-management. In this study, the scale showed acceptable reliability, with a Cronbach’s *α* of 0.818, consistent with prior reports of a full-scale α of 0.914 and subscale α values ranging from 0.3 to 0.8 (13).

#### 2.3.4 Self-efficacy scale for hypertensive patients

Self-efficacy in hypertension management was evaluated with a scale developed by YANG Bi-ping to assess patients’ confidence in managing their condition (14). This 11-item instrument covers four dimensions: daily life behaviors (3 items), medication adherence (3 items), health behaviors (2 items), and adherence to medical advice (3 items). Each item is scored from 0 (“not at all”) to 4 (“extremely”), resulting in a total score between 0 and 44. Higher scores indicate greater self-efficacy. In the current study, the scale exhibited good internal consistency, with a Cronbach’s *α* of 0.803, comparable to previously reported values of 0.80 for the full scale and 0.641–0.871 for its subscales (14).

#### 2.3.5 Health literacy management scale, HeLMS

Health literacy was assessed using the Chinese version of a scale originally developed by Jordan et al. and cross-culturally adapted by Sun Haolin (15, 16). This instrument evaluates the abilities and cognitions needed to obtain, understand, and use health information and services. It includes 24 items across four dimensions: information acquisition ability (9 items), communication and interaction ability (9 items), willingness to improve health (4 items), and willingness to secure economic support (2 items). Responses are given on a 5-point Likert scale (1 = “not at all” to 5 = “extremely”), with total scores ranging from 24 to 120. Higher scores indicate better health literacy. In this study, the scale demonstrated good reliability, with a Cronbach’s *α* of 0.814, aligning with earlier reports of a full-scale α of 0.894 and subscale α values between 0.495 and 0.813 for the Chinese version (16).

### 2.4 Statistical analysis

Data entry was performed using Epidata 3.1 software, and statistical analyses were conducted using SPSS 27.0. Continuous variables were presented as the mean and standard deviation, while categorical variables were described as frequencies and percentages. Differences in sociodemographic and clinical characteristics across the identified latent classes were examined using one-way analysis of variance (ANOVA) for continuous variables and the Chi-square test for categorical variables. Subsequently, variables that showed statistical significance in these univariate analyses were entered as independent variables into a multinomial logistic regression model, with the latent profiles as the dependent variable, to identify factors associated with class membership. A two-sided *p*-value < 0.05 was considered statistically significant.

Latent profile analysis (LPA) was performed in Mplus 8.3 to identify distinct subtypes of proactive health behavior, using scores from all dimensions of the Proactive Health Behavior Scale for Hypertensive Patients. Models specifying one to five latent profiles were estimated and compared. Model fit was evaluated using the following indices: the Akaike Information Criterion (AIC), Bayesian Information Criterion (BIC), sample-size adjusted BIC (aBIC), entropy, the Lo–Mendell–Rubin adjusted likelihood ratio test (LMRT), and the bootstrap likelihood ratio test (BLRT).

The lower values of AIC, BIC, and aBIC, the more accurate the classifcation of the model (17). Entropy represents the accuracy of classifcation ranging between 0 and 1. With values of 0.80 or higher indicates a classifcation accuracy exceeding 90% or more (18). LMRT and BLRT based on bootstrap were used to assess the fit diferences between n-1 and n profile models. A *p* value less than 0.05 indicates that a n model signifcantly outperforms a n-1 model (19). A thorough evaluation of all indices is essential to determine the most optimal model (17).

### 2.5 Ethical considerations

This study was conducted in accordance with the ethical standards of the institution and was approved by the Ethics Committee of Anhui University of Chinese Medicine (Approval No. AHUCM-HSS-2025008). All participants provided written informed consent after being fully informed of the study’s purpose and procedures. The survey was conducted anonymously, and no clinical interventions were involved. To ensure confidentiality, all questionnaires were labeled with unique identification codes, and electronic data were stored on encrypted, password-protected servers. The study involved no more than minimal risk to participants.

## 3 Results

### 3.1 Demographic and clinical characteristics

A total of 352 questionnaires were distributed, yielding 321 valid responses (effective response rate: 91.2%). Among the participants, 155 (48.3%) were female and 166 (51.7%) were male; 110 (34.3%) were aged <45 years, 122 (38.0%) were 45–59 years, and 89 (27.7%) were ≥60 years. A total of 126 participants (39.3%) held a bachelor’s degree or higher. Regarding hypertension severity, 155 patients (48.3%) were classified as Stage I, 135 (42.1%) as Stage II, and 31 (9.7%) as Stage III. Detailed demographic and clinical characteristics, along with results of the univariate analyses, are presented in Table 1.

**Table 1:** Demographic characteristics of patients with hypertension and their single-factor analysis on proactive health behavior profles [mean ± SD or n (%)].

Variables		Overall(*n* = 321)	Class 1: Positive proactive health behavior type (*n* = 50)	Class 2: Self-regulating proactive health behavior type (*n* = 114)	Class 3: Medically Compliant-proactive health behavior type (*n* = 96)	Class 4: Passive proactive health behavior type (*n* = 61)	F/X^2^	*p* value
Gender	Male	166(51.7%)	24(48.0%)	63(55.3%)	52(54.2%)	27(44.3%)	2.439	0.486
	Female	155(48.3%)	24(52.0%)	51(44.7%)	44(45.8%)	34(55.7%)		
Age(year)	<45	110(34.3)	23(46.0)	50(43.9)	27(28.1)	10(16.4)	18.942	0.004
	45–59	122(38.0)	18(36.0)	37(32.5)	38(39.6)	29(47.5)		
	≥60	89(27.7)	9(18.0)	27(23.7)	31(32.3)	22(36.1)		
Education	Senior high school and below	195(60.7)	31(62.0)	71(62.3)	44(45.8)	49(80.3)	18.908	<0.001
	Bachelor’s degree and above	126(39.3)	19(38.0)	43(37.7)	52(54.2)	12(19.7)		
Marital status	Married	253(78.8)	32(64.0)	89(78.1)	85(88.5)	44(77.0)	12.164	0.007
	Single or others	68(21.2)	18(36.0)	25(21.9)	11(11.5)	14(23.0)		
Employment status	Yes	207(64.5)	19(38.0)	85(74.6)	62(64.6)	41(67.2)	20.567	<0.001
	No	114(35.5)	31(62.0)	29(25.4)	34(35.4)	20(32.8)		
Smoking history	Yes	104(32.4)	16(32.0)	40(35.1)	27(28.1)	21(34.4)	1.295	0.730
	No	217(67.6)	34(68.0)	74(64.9)	69(71.9)	40(65.6)		
Drinking history	Yes	93(29.0)	14(28.0)	35(30.7)	27(28.1)	17(27.9)	0.258	0.968
	No	228(71.0)	36(72.0)	79(69.3)	69(71.9)	44(72.1)		
BMI^1^	Underweight	53(16.5)	9(18.0)	20(17.5)	14(14.6)	10(16.4)	1.96	0.923
	Normal	224(69.8)	35(70.0)	75(65.8)	70(72.9)	44(72.1)		
	Overweight/obese	44(13.7)	6(12.0)	19(16.7)	12(12.5)	7(11.5)		
Disease duration (year)	<5	176(54.8)	10(16.4)	81(71.1)	37(38.5)	36(72.0)	63.745	<0.001
	5≤10	108(33.6)	37(60.7)	25(21.9)	40(41.7)	8(16.0)		
	≥10	37(11.5)	14(23.0)	8(7.0)	19(19.8)	6(12.0)		
Classification of hypertension^2^	I	155(48.3)	27(44.3)	57(50.0)	49(51.0)	22(44.0)	6.917	0.329
	II	135(42.1)	23(37.7)	49(43.0)	40(41.7)	23(46.0)		
	III	31(9.7)	11(18.0)	8(7.0)	7(7.3)	5(10.0)		
Hospitalization due to hypertension	Yes	170(53.0)	20(32.8)	79(69.3)	34(35.4)	34(68.0)	38.395	<0.001
	No	151(47.0)	41(67.2)	35(30.7)	62(64.6)	16(32.0)		
Hypertension-related comorbidities	Yes	107(33.3)	24(39.3)	35(30.7)	30(31.3)	18(36.0)	1.695	0.638
	No	214(66.7)	37(60.7)	79(69.3)	66(68.8)	32(64.0)		
Self-management level (score range 33–165)	-	96.74 ± 23.94	79.72 ± 15.73	102.77 ± 22.09	99.02 ± 25.69	99.38 ± 23.95	14.896	<0.001
Self-efficacy level (score, range 0–44)	-	27.12 ± 8.76	20.80 ± 7.53	29.46 ± 8.64	26.98 ± 8.16	29.78 ± 7.78	17.066	<0.001
Health literacy level (score, range 24–120)	-	72.37 ± 23.70	79.67 ± 22.29	73.68 ± 17.93	71.47 ± 23.79	59.07 ± 24.87	11.057	<0.001

1 BMI categories were defined as follows (49): underweight (<18.5 kg/m^2^), normal (18.5–23.9 kg/m^2^), overweight (24.0–27.9 kg/m^2^), and obese (≥28.0 kg/m^2^).

2 Hypertension classification were graded according to the Chinese Guidelines (12) as: Grade I (systolic blood pressure [SBP] 140–159 mmHg or diastolic blood pressure [DBP] 90–99 mmHg), Grade II (SBP 160–179 mmHg or DBP 100–109 mmHg), and Grade III (SBP ≥ 180 mmHg or DBP ≥ 110 mmHg; 1 mmHg = 0.133 kPa).

### 3.2 Model selection

The score of proactive health behaviors among 321 patients with hypertension ranged from 38 to 142 (89.57 ± 22.99). Based on the scores of five dimensions of proactive health behavior among patients with hypertension, one to five latent profile models were sequentially fitted, shown in Table 2.

**Table 2:** Model fitting information for the latent profile of patients’ proactive health behavior (*n* = 321).

Model	AIC	BIC	aBIC	Entropy	*p* value		Class probability (%)
					LMRT	BLRT	
1-Profle	4487.945	4525.659	4493.941	—	—	—	—
2-Profle	4064.193	4124.536	4073.787	0.949	<0.001	<0.001	82.5/17.5
3-Profle	3858.781	3941.753	3871.973	0.868	<0.001	<0.001	43.0/41.1/15.9
4-Profle	3741.096	3846.697	3757.885	0.856	<0.001	<0.001	30.2/18.6/15.8/35.4
5-Profle	3727.991	3856.22	3746.377	0.821	0.301	<0.001	19.0/26.2/27.7/11.3/15.8

aBIC, sample size-adjusted Bayesian information criterion; LMR, Lo–Mendell–Rubin adjusted likelihood ratio test; AIC, Akaike information criterion; BIC, Bayesian information criterion; BLRT, bootstrap likelihood ratio test. *p* < 0.05: The n-category model is significantly superior to the n-l-category model.

Regarding information criteria, the values of AIC, BIC, and aBIC generally decreased as the number of classes increased. By the 4-class model, the decline showed a clear moderating trend. Although the 5-class model exhibited slightly lower AIC and aBIC values compared to the 4-class model, the BIC value increased (3846.697 vs. 3856.220). Since BIC imposes a stricter penalty on model complexity, its rise suggests that the limited improvement in fit gained by adding a fifth class does not justify the increased model complexity, indicating a risk of overfitting.

In terms of classification accuracy, the entropy values for the 2- to 5-class models all fell within the acceptable range (above 0.8). Although the entropy of the 4-class model was lower than that of the 2- and 3-class models, it remained relatively high and was significantly greater than that of the 5-class model. This indicates that the 4-class model ensures both clarity in sample classification and adequate representation of heterogeneity across classes.

The likelihood ratio tests showed that both the LMRT and BLRT supported the appropriateness of the 2- to 4-class models. In contrast, the LMRT *p*-value for the 5-class model was 0.301 (*p* > 0.05), suggesting no statistically significant difference between the 5-class and 4-class models. Thus, adding a fifth class does not further optimize model fit.

From the perspective of class distribution and interpretability, the smallest class in the 4-class model accounted for 15.8% of the sample, with balanced proportions across classes (ranging from 15.8 to 35.4%), which facilitates subsequent clinical interpretation and the development of targeted intervention strategies. In comparison, the 5-class model included a relatively small class comprising only 11.3% of the sample, which may lead to unstable class characteristics and complicate interpretation.

Following a comprehensive evaluation of the model fit indices, the 4-profile model was found to be the optimal model for interpretation and additional analysis.

### 3.3 Characteristics and naming of latent profiles of proactive health behavior in hypertension patients

**Figure 1:**
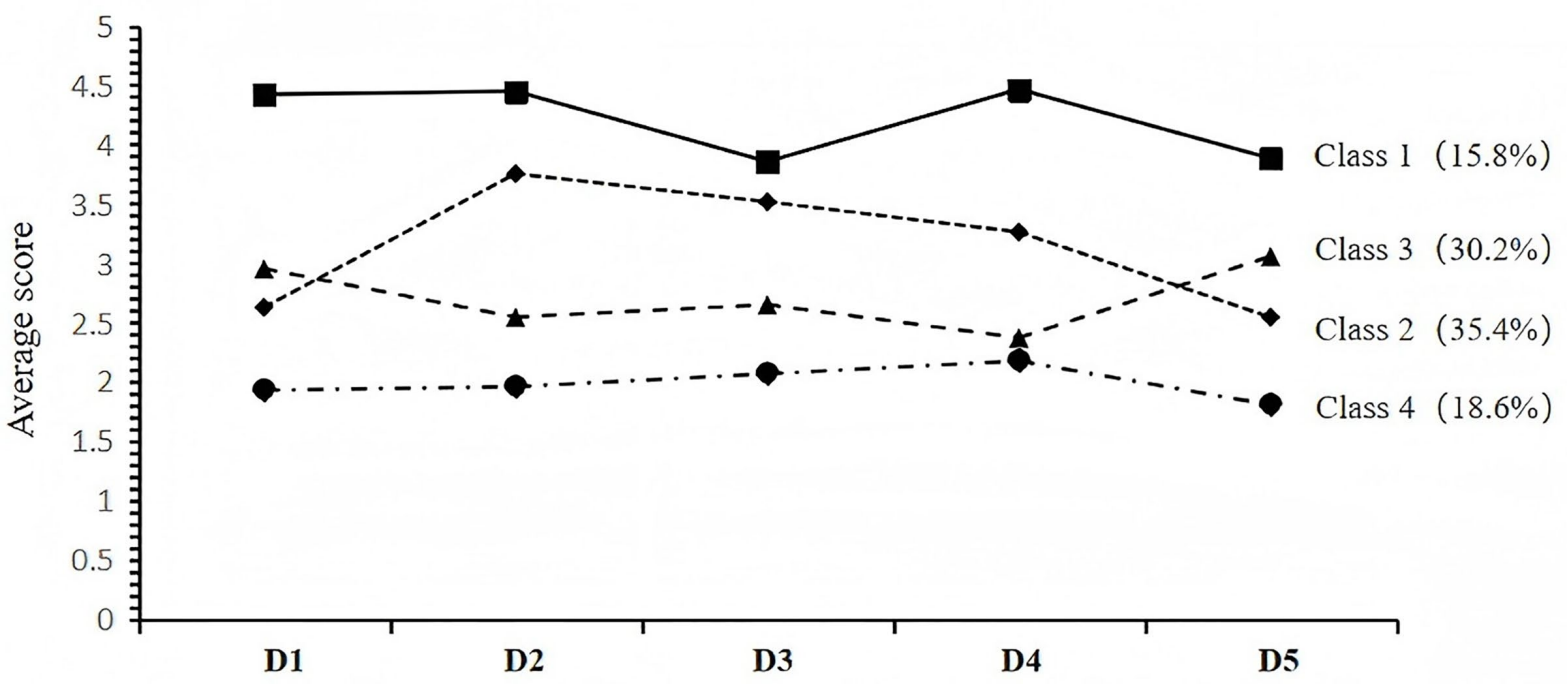
Four-category latent profile analysis proactive health behavior in patients with hypertension. Horizontal axis: 5 dimensions of the Proactive Health Behavior Scale (patients with hypertension); D1 = self-health responsibility, D2 = dietary management, D3 = physical activity management, D4 = labor and emotional management, D5 = disease management.

Latent Profile Analysis (LPA) was conducted to identify subgroups of proactive health behaviors among patients with hypertension, with the final four-class model presented in Figure 1: Four-Category Latent Profile Analysis of Proactive Health Behaviors in Patients with Hypertension. Based on the characteristic patterns of each latent class, distinct nomenclatures were assigned accordingly.

Class 1: This profile includes 50 patients (15.8%) with the highest proactive health behavior score of 130.68 ± 6.15. This cluster was characterized by a high sense of self-health responsibility (ranked first in the self-health responsibility dimension) and proactive health behaviors (ranked first in dimensions including diet, exercise, work, emotional management, and disease management). It was thus termed the “Positive-Proactive Health Behavior Profile.”

Class 2: This cluster was the largest, comprising 114 patients (35.4%), with a proactive health behavior score of 95.04 ± 8.12 and ranked second in total score among the four clusters. This cluster was characterized by self-regulating practices in daily health behaviors (ranked second in dimensions of dietary, physical activity, labor and emotional management), yet demonstrated insufficient motivation for self-health responsibility (ranked third) and suboptimal performance in disease management (ranked third). It was consequently designated as the “Self-regulating-Proactive Health Behavior Profile.”

Class 3: This cluster comprised 96 patients (30.2%), with a proactive health behavior score of 80.49 ± 8.05 points and ranked third in total score among the four clusters. Patients in this cluster demonstrated relatively good motivation and adherence within the bounds of medical compliance (ranked second in both self-health responsibility and disease management dimensions), yet performed poorly in self-regulating daily practices (ranked third in dimensions such as dietary, physical activity, labor and emotional management). Such a pattern of proactive behavior is mainly attributed to driving factors stemming from medical compliance. Therefore, they are named as “Medically Compliant-proactive health behavior Profile.”

Class 4: This profile comprised 61 patients (18.6%), scoring the lowest in proactive health behavior (59.92 ± 8.32) and ranked last across all dimensional scores. These patients were characterized by a pervasive lack of self-health responsibility and marked passivity and negligence in both disease management and daily health behaviors, and were thus termed the “Passive-Proactive Health Behavior Profile.”

### 3.4 Results of multinomial logistic regression of the latent profile of proactive health behaviors in hypertensive patients

The multinomial logistic regression model incorporated variables that demonstrated statistical significance across the four profiles in Table 1. The covariates comprised scores for self-management, self-efficacy, and health literacy. The categorical variables included age, educational level, marital status, employment status, disease duration, and history of hospitalization for hypertension. The variable assignments are presented in Table 3.

**Table 3:** Variable assignment.

Variable	Assignment explanation
1. Age (years)	<45 = 1, 45–59 = 2, ≥ 60 = 3
2. Educational level	bachelor’s degree and above = 2, senior high school and below = 1
3. Marital status	Yes = 1, No = 2
4. Employment status	Yes = 1, No = 2
5. Disease duration	<5 = 1, 5≤10 = 2, ≥10 = 3
6. Hospitalization due to hypertension	Yes = 1, No = 2
7. Self-management	Actual data
8. Self-efficacy	Actual data
9. Health literacy	Actual data

As shown in Table 4, multiple factors were significantly associated with different proactive health behavior profiles, using the “Passive-Proactive Profile” as the reference.

**Table 4:** Multinomial logistic regression analysis of variables influencing proactive health behaviors profiles.

Variables		Self-regulating-proactive profile (vs. passive-proactive profile)					Medically compliant-proactive profile (vs. Passive-proactive profile)					Positive-proactive profile (vs. passive-proactive profile)				
		*β*	Odds ratio	*p* value	95%CI		*β*	Odds ratio	*p* value	95%CI		*β*	Odds ratio	*p* value	95%CI	
					Lower	Upper				Lower	Upper				Lower	Upper
Age(year)	<45	0.861	2.365	0.238	0.566	9.893	0.544	1.723	0.442	0.431	6.885	3.061	21.352	<0.001	4.026	113.254
	45–59	0.189	1.209	0.763	0.353	4.133	0.178	1.195	0.758	0.384	3.716	2.102	8.183	0.004	1.96	34.16
	≥60(ref.)	-	-	-	-	-	-	-	-	-	-	-	-	-	-	-
Educational level	Senior high school and below	−0.661	0.516	0.173	0.199	1.337	−1.534	0.216	0.001	0.088	0.527	−0.688	0.503	0.215	0.169	1.492
	Bachelor’s degree and above(ref.)	-	-	-	-	-	-	-	-	-	-	-	-	-	-	-
Marital status	Yes	0.173	1.189	0.739	0.43	3.291	1.072	2.92	0.043	1.033	8.255	−0.604	0.547	0.296	0.176	1.698
	No(ref.)	-	-	-	-	-	-	-	-	-	-	-	-	-	-	-
Employment status	Yes	−0.363	0.696	0.56	0.205	2.359	−0.798	0.45	0.167	0.145	1.398	−2.854	0.058	<0.001	0.015	0.217
	No(ref.)	-	-	-	-	-	-	-	-	-	-	-	-	-	-	-
Disease duration(year)	<5	2.295	9.927	<0.001	2.844	34.656	0.757	2.131	0.185	0.695	6.533	1.56	4.759	0.027	1.195	18.958
	5–10	0.397	1.487	0.515	0.451	4.898	−0.128	0.88	0.797	0.33	2.343	−0.448	0.639	0.537	0.154	2.651
	≥10(ref.)	-	-	-	-	-	-	-	-	-	-	-	-	-	-	-
Hospitalization due to hypertension	Yes	1.142	3.133	0.009	1.323	7.423	−0.182	0.833	0.669	0.362	1.92	0.865	2.374	0.094	0.863	6.534
	No(ref.)	-	-	-	-	-	-	-	-	-	-	-	-	-	-	-
Self-efficacy (score)		0.122	1.129	<0.001	1.072	1.19	0.101	1.107	<0.001	1.052	1.164	0.114	1.121	<0.001	1.054	1.192
Self-management (score)		0.04	1.041	<0.001	1.019	1.064	0.034	1.034	0.001	1.013	1.056	0.036	1.037	0.004	1.011	1.063
Health literacy (score)		0.03	1.03	<0.001	1.012	1.049	0.019	1.02	0.025	1.002	1.037	0.017	1.018	0.108	0.996	1.039

For the Self-Regulating-Proactive Profile, positive predictors included a disease duration of <5 years (OR = 9.927, 95% CI: 2.844–34.656), hospitalization due to hypertension (OR = 3.133, 95% CI: 1.323–7.423) as well as higher scores in self-management (OR = 1.041, 95% CI: 1.019–1.064), self-efficacy (OR = 1.129, 95% CI: 1.072–1.19), and health literacy (OR = 1.03, 95% CI: 1.012–1.049).

For the Medically Compliant-Proactive Profile, positive predictors included married status (OR = 2.92, 95% CI: 1.033–8.255), higher self-management (OR = 1.034, 95% CI: 1.013–1.056), and self-efficacy (OR = 1.107, 95% CI: 1.052–1.164). Patients with senior high school and below were a negative predictor of the Medically Compliant-Proactive Profile (OR = 0.216, 95% CI: 0.088–0.527).

For the Positive-Proactive Profile, positive predictors included younger age (<45 years: OR = 21.352, 95% CI: 4.026–113.254; 45–59 years: OR = 8.183, 95% CI: 1.96–34.16), a disease duration of <5 years(OR = 4.759, 95% CI: 1.195–18.958), and higher scores in self-management (OR = 1.037, 95% CI: 1.011–1.063), self-efficacy (OR = 1.121, 95% CI: 1.054–1.192). Being employed is a negative predictor of the Positive-Proactive Profile (OR = 0.058, 95% CI: 0.015–0.217).

## 4 Discussion

The study revealed significant heterogeneity in proactive health behaviors among hypertension patients, which could be categorized into four distinct profiles, underscoring the need for classification-specific precision management of blood pressure.

### 4.1 Overall level and profile characteristics of proactive health behaviors in hypertension patients

In this study, the total score for patients’ proactive health behaviors was 89.57 ± 22.99. Relative to the scale midpoint of 90 (6), this reflects a moderate level, though it remains significantly lower than that reported among hospitalized hypertension patients (109.88 ± 6.82) (20). From the perspective of HBM, hospitalized patients are consistently exposed to external cues to action, such as ongoing medical supervision and structured health education, which enhance health literacy, strengthen perceived susceptibility and severity of the disease, and reduce perceived barriers through systematic support. Positive and immediate feedback on blood pressure control further reinforces perceived benefits and self-efficacy, thereby contributing to higher proactive health behavior scores. In contrast, this study focused on community-dwelling hypertension patients. Their relatively stable blood pressure and limited external cues in the community setting may weaken perceived severity and susceptibility to the disease. Additionally, substantial individual differences in the ability to cope with self-management barriers lead to generally lower levels of proactive health behaviors compared to hospitalized patients, along with more pronounced interindividual heterogeneity.

The Positive-Proactive Profile was characterized by the highest scores in proactive health behaviors. From the perspective of HBM, this group demonstrated a highly internalized health belief system (the dimension of self-health responsibility ranked first among the four profiles). They showed clear awareness of the perceived susceptibility to and severity of hypertension, accurately recognized the long-term benefits of consistent disease management, and exhibited strong self-efficacy in overcoming barriers (the dimension of disease management ranked first). This has established a positive belief–behavior cycle, as reflected in their top rankings in the dimensions of dietary, physical activity, labor, and emotional management. Nevertheless, this group accounted for only 15.8% of the sample, indicating that patients with a high level of proactive health management capability remain a minority.

The Self-Regulating-Proactive Profile constituted the largest subgroup (35.4%), representing the predominant behavioral pattern in this hypertensive cohort. This group exhibited consistent self-regulation in daily health practices, with scores ranked second across dietary, physical activity, labor and emotional management dimensions. In contrast, they showed diminished personal health responsibility (ranked third) and lower engagement in disease management (ranked third). From a HBM perspective, this profile reflects a structural divergence in perceived health benefits: participants recognized and actively adopted self-regulated daily health behaviors, yet underappreciated the long-term benefits of medically guided health actions. These medically oriented behaviors were also more vulnerable to perceived barriers, including time, cost, and medication-related concerns, further undermining adherence. Thus, this pattern illustrates a selective behavioral orientation characterized by proactive self-regulation in lifestyle, alongside suboptimal compliance with medical management.

The Medically Compliant-Proactive Profile accounted for 30.2% of the sample and was characterized by relatively clear self-health responsibility (ranked second). In structured disease management settings, external behavioral cues, such as reminders from healthcare professionals and standardized management protocols, interacted with patients’ pre-existing health beliefs, driving them to comply with disease management regimens (ranked second in the disease management dimension). However, their health beliefs were not fully internalized. When returning to unstructured daily life scenarios lacking external guidance or constraints, these patients exhibited increased perceived barriers to proactive health behaviors and diminished perceived benefits. Their self-regulatory capacity concerning diet, exercise, labor and emotion was comparatively limited (ranking third in each respective dimension). Overall, their behavioral pattern is characterized by compliance-oriented pattern in health management.

Passive-Proactive Profile accounted for 18.6% of the sample and exhibited the lowest scores across all dimensions of proactive health behavior. Patients in this profile demonstrated low awareness of self-health responsibility. Beyond maintaining a basic balance between labor and emotional management, they showed passive or minimal engagement in dietary control, physical activity, and disease management. Overall, their health behavior agency was the weakest, characterized by a systematically deficient health belief system that resulted in insufficient motivation to initiate and sustain health-promoting behaviors.

### 4.2 Differential impact of multiple factors on the potential categories of proactive health behaviors

The analysis revealed divergent patterns of influence exerted by demographic, clinical, and psychological factors on different proactive health behavior profiles, a finding that precisely underscores the value of the latent profile approach.

Regarding demographic factors, age emerged as a significant predictor of the Positive-Proactive Profile. Patients aged <45 years had 21.352 times higher odds (95% CI: 4.026–113.254) of belonging to this profile compared to those aged ≥60 years. Patients aged 45–59 years also had significantly higher odds (OR = 8.183, 95% CI: 1.96–34.16). This gradient aligns with theoretical expectations. Drawing on Social Cognitive Theory (21) and the Health Belief Model, younger individuals typically exhibit superior information processing and self-regulatory skills, enabling them to better perceive health threats, appraise the long-term benefits of proactive behaviors, and convert intentions into action. Conversely, older adults may face elevated perceived barriers due to age-related cognitive changes and challenges in engaging with digital health tools, which can weaken their responsiveness to behavioral cues. This combination of factors may predispose them toward more passive behavioral profiles, such as the Passive-Proactive Profile identified in this study (22).

Employment status was a significant negative predictor of the Positive-Proactive Profile (OR = 0.058, 95% CI: 0.015–0.217), indicating that the workplace context and occupational stress may constrain proactive health behaviors. On one hand, the typical work environment, characterized by prolonged sitting and frequent dining out, limits opportunities for physical activity and healthy eating, thereby increasing perceived barriers to behavior change. On the other hand, occupational stress elevates cortisol levels, which activates the sympathetic nervous system and exacerbates blood pressure fluctuations. This physiological response makes it difficult for individuals to perceive the tangible benefits of their health efforts, thereby weakening their perceived benefits. Furthermore, sustained work demands deplete psychological resources, leading to diminished self-efficacy in maintaining health behaviors. When combined with low health literacy, this depletion further impairs an individual’s ability to accurately appraise the perceived benefits and barriers associated with behavior change (23, 24). It is worth further exploring that the mechanisms of influence of different occupational types on health behaviors may vary. For example, although manual workers expend considerable physical energy in their jobs, their work may lead to irregular routines and physical fatigue, thereby reducing their motivation for proactive exercise. In contrast, white-collar workers are more susceptible to prolonged sedentary behavior and high cognitive demands, making it challenging for them to maintain regular health behaviors. Future studies could collect detailed occupational information (e.g., job nature, intensity, and environment) to thoroughly examine the pathways through which occupational factors influence the health behaviors of hypertensive patients, thereby providing a basis for developing targeted health interventions for specific occupational groups.

Marital status and educational level emerged as significant predictors specifically for the Medically Compliant-Proactive Profile. Although marital status is traditionally considered a protective factor, this study found that married patients had 2.92 times higher odds (95% CI: 1.033–8.255) of belonging to the Medically Compliant-Proactive Profile compared to the Passive-Proactive Profile, yet it did not increase their likelihood of belonging to the Positive-Proactive Profile. This dual effect can be interpreted through two complementary mechanisms. The supportive function of marriage is reflected in the spouse often serving as an informal health supervisor, helping the patient maintain compliance with disease management within the home setting. Furthermore, as a central component of the social support system, marriage alleviates the perceived burden of disease management through practical assistance, such as facilitating healthcare access and offering emotional support, thereby reducing passive tendencies arising from loneliness or practical challenges. Conversely, from the perspective of familial responsibility structures common in Chinese society (25), marital and domestic obligations (e.g., spousal care, child-rearing) compete directly with the substantial time and cognitive resources required for more complex, self-initiated health behaviors, such as regular exercise, deliberate meal planning, and autonomous stress management. This competition for personal resources explains why marriage, while effectively supporting externally guided compliance (as seen in the Medically Compliant-Proactive Profile), may not sufficiently promote the internalization of health motivations or the broad behavioral expansion necessary for progression to the Positive-Proactive Profile.

Educational level was also a significant predictor of profile membership. Compared to patients with a college education or above, those with a high school education or less had significantly higher odds of belonging to the Passive-Proactive Profile relative to the Medically Compliant-Proactive Profile (OR = 0.216, 95% CI: 0.088–0.527). This pattern may stem from limited health literacy, which can constrain access to health information, impair comprehension of medical advice, and exacerbate practical barriers to behavior change. These factors collectively reduce both the motivation to adopt healthy behaviors and the perceived benefits of engaging with formal healthcare support. Consequently, under conditions of low perceived benefits and high perceived barriers, their behavioral patterns are more likely to remain within the passive range.

Regarding clinical factors, a disease duration of <5 years was a significant positive predictor for both the Self-Regulating-Proactive Profile (OR = 9.927, 95% CI: 2.844–34.656) and the Positive-Proactive Profile (OR = 4.759, 95% CI: 1.195–18.958), with a notably higher probability of belonging to the Self-Regulating-Proactive Profile. From the perspective of behavioral development, the consolidation of health behaviors requires sustained repetition and positive reinforcement. Patients with shorter disease duration are typically in a phase of behavioral adjustment (26, 27), which represents a critical transitional period toward proactive health engagement. According to the HBM, whether patients successfully navigate this transition depends on the interplay of multiple factors, including perceived disease threat, evaluation of benefits versus barriers to behavior change, and access to cues for action. Consequently, not all patients achieve a smooth transition to sustained proactive behavior. This also explains why, in the present study, patients with a disease duration of <5 years were more likely to be classified into the Self-Regulating-Proactive Profile than into the Positive-Proactive Profile.

A history of hospitalization significantly increased the odds of transitioning from the Passive-Proactive to the Self-Regulating-Proactive Profile (OR = 3.133, 95% CI: 1.323–7.423). This may be explained by the intensive health education provided during hospitalization, which serves as a powerful external cue to action (28), which likely enhances patients’ perceived severity of hypertension. Simultaneously, the immediate feedback from improved blood pressure control reinforces their perceived benefits of behavioral change, thereby facilitating the shift toward more proactive self-management.

Both self-efficacy and self-management capacity were significant negative predictors of the Passive-Proactive Profile. As a core dimension of the HBM, self-efficacy functions as a pivotal mediator, translating perceived benefits into sustained health action. Individuals with higher self-efficacy are more confident in their ability to overcome barriers and to derive tangible benefits from health behaviors, which promotes movement away from passive patterns (29). Similarly, greater self-management capacity reduces perceived barriers (30) by enabling consistent execution of key behaviors such as blood pressure monitoring and dietary control. Successful hypertension management then reinforces perceived benefits, establishing a positive feedback loop that supports ongoing engagement. Conversely, patients with limited self-management skills often experience poor behavioral execution and inadequate blood pressure control. This not only interrupts the reinforcement of perceived benefits but also amplifies perceived barriers, thereby increasing the likelihood of remaining in a passive behavioral state.

Health literacy positively predicted membership in both the Self-Regulating-Proactive Profile (OR = 1.030, 95% CI: 1.012–1.049) and the Medically Compliant-Proactive Profile (OR = 1.020, 95% CI: 1.002–1.037), but was not significantly associated with the Positive-Proactive Profile. This differential pattern can be explained by the interplay between behavioral development stages and a functional threshold effect. First, health literacy primarily facilitates the translation of health information into internalized beliefs, a process especially critical for patients in the cognitive and behavioral adjustment phases, as typified by the Self-Regulating and Medically Compliant profiles. In contrast, individuals in the Positive-Proactive Profile have largely completed the transition from cognitive appraisal to belief internalization; thus, the supportive function of health literacy may be supplanted by higher-order, self-sustaining mechanisms that maintain established proactive routines. Second, health literacy appears subject to a threshold effect (31). Members of the Positive-Proactive Profile likely already possess health literacy at or above a functional saturation point, where further increments yield diminishing returns on behavior maintenance. Conversely, for those in the Self-Regulating and Medically Compliant profiles, health literacy generally remains below this threshold, so even modest improvements can substantially enhance information comprehension and belief consolidation, thereby producing a more pronounced and statistically detectable predictive relationship.

### 4.3 Nursing implications

Patients with a Positive-Proactive Profile are characterized by stable health beliefs and robust behavioral execution capacity. Shared care clinics, a group-based care model accommodating 10–15 patients per session (32), can serve as a platform to engage these patients as peer influencers. By sharing their personal experience and success stories, they can provide concrete cues to action for patients with other behavioral profiles, helping the latter directly perceive the tangible benefits of positive health behaviors. Moreover, their replicable practical examples can effectively reduce the perceived barriers to behavior change among these patients. Meanwhile, the multidisciplinary support available in shared care clinics can deliver advanced health guidance for Positive-Proactive Profile patients, thereby preventing burnout induced by long-term self-management. Developing stratified, context-sensitive interventions requires dedicated efforts to address the contextual needs of distinct patient subgroups. For employed patients, fragmented, context-adapted health management strategies are recommended, such as Sheetali and Bhramari pranayama breathing techniques (33) and autonomous AI-driven digital health interventions (34). For older adults patients, it is imperative to develop age-friendly intervention tools to minimize implementation barriers, including blood pressure monitors equipped with voice guidance and illustrated dietary manuals (35). The VITASENIOR-MT platform exemplifies an effective practice in this regard (36). It adopts a television-based interface that is familiar to the older adults for data entry, which lowers the threshold for technology adoption. Additionally, it leverages Internet of Things (IoT) technology to enable automatic data transmission, thereby significantly improving older adults users’ engagement and treatment adherence.

For patients with the Self-Regulating-Proactive Profile, real-world case-based visual materials (e.g., images of organ damage induced by hypertension) (37, 38) and hypertension-related complication risk calculation models (39, 40) can be adopted to enhance their intuitive understanding of disease risks and motivate them to engage in health management. Meanwhile, personalized reminders can be delivered via text messages, mobile applications and other channels. In combination with regular follow-ups or peer support groups, positive feedback should be provided on their behavioral progress, so as to gradually improve their self-efficacy and behavioral execution capacity. For patients with a short disease course, interventions targeting habit formation should be strengthened. Repetitive action cues can be used to facilitate behavioral habituation and prevent regression to a passive state. Drawing on the Discharge Transition Care Theory (41), the period from pre-discharge to 2 months post-discharge represents a golden window for consolidating patients’ health beliefs. During this period, continuous external cues can be provided through remote monitoring and systematic follow-ups to maintain patients’ high-level disease awareness, thereby promoting their transition to the Positive-Proactive Profile. The virtual visit platform established by Levine et al. (42) for hypertensive patients has shortened consultation time and achieved high patient satisfaction. This indicates that such digital tools can serve as effective carriers for reducing perceived barriers and providing action cues, supporting patients in consistently practicing healthy behaviors in daily life scenarios.

For patients with the Medically Compliant-Proactive Profile, within the family support system, efforts should be made to facilitate the transformation of spouses’ role from informal health managers to co-participants. Through collaborative approaches such as accompanying patients in physical exercises and providing emotional mutual support, spouses can practice health behaviors together with patients in daily life (43). In addition, collective family tasks such as preparing low-salt meals together and outdoor activities can be organized to naturally integrate health management into daily routines, promote the organic combination of health behaviors and family responsibilities, and further facilitate the internalization and sustained practice of health behaviors among patients. To achieve the above goals of collaborative support and behavioral integration, Huilong Duan et al. (44) developed a set of digital health intervention tools based on the concept of Goal-Directed Design. Built on a mobile health application framework, this tool incorporates personalized goal-setting and family collaboration modules, thereby strengthening family-centered health behavior support and implementation. Based on the Behavior Change Wheel theory, Wei Gan et al. designed an Interactive Pictorial Health Education (IPHE) program targeting patients with low literacy levels. By visualizing complex medical information through graphic illustrations, this program enhances the understanding and engagement of patients with limited literacy (45).

For patients with the Passive-Proactive Profile, the core of interventions lies in stimulating their intrinsic health motivation and establishing a sustainable health belief system. To achieve this goal, the concept of Patient Activation can be introduced, which serves as a predictive indicator for measuring an individual’s willingness and ability to engage in personal health management (46). In the future, this concept can be embedded into personalized health education delivery algorithms to enable precise matching and dynamic adaptation of supportive content for patients. On the basis of motivation cultivation, the focus of interventions should shift to reducing patients’ perceived barriers, as well as improving their self-efficacy, self-management capacity and health literacy. Chen P. developed an artificial intelligence-powered conversational agent that constructs a personalized health knowledge base using knowledge graphs (47), allowing patients to access accurate information and receive instant answers anytime and anywhere. This process not only optimizes patients’ health literacy, but also accumulates successful experiences through continuous positive interactions, thereby enhancing their self-efficacy and self-management capacity. Furthermore, the data visualization capabilities of wearable devices (48) can be incorporated into interventions. This allows patients to directly observe the subtle health improvements resulting from behavioral changes, using visual evidence to counteract negative perceptions.

## 5 Limitations

Although this study provides practical insights into the proactive health behaviors of hypertensive patients, several limitations should be acknowledged. First, its cross-sectional design can reveal correlations but cannot establish causality or track dynamic evolution. Second, it did not measure specific community health support indicators (e.g., family doctor contracting rates, health lecture participation), limiting the analysis of how external systems moderate different proactive behaviors. Third, although the sample of this study was drawn from eight communities in Anhui Province, covering both urban and rural settings, it still carries geographic limitations. Future research could adopt a cross-regional, multi-center sampling design to further examine the stability of the identified latent profiles and explore how factors such as economic development levels, regional differences in health beliefs, and coverage of public health services may contribute to heterogeneity in influencing factors.

## 6 Conclusion

This study used latent profile analysis to identify four distinct subtypes of proactive health behaviors among community-dwelling hypertensive patients and systematically analyzed the differential factors associated with each subtype from the perspective of the HBM. This typological perspective moves beyond describing overall behavioral levels and enriches our understanding of the complexity inherent in patients’ proactive health behaviors. The findings suggest that health promotion interventions need to move beyond a one-size-fits-all approach. Instead, they should integrate individual, family, and digital technology resources to implement strategies targeted at patients’ specific behavioral subtypes, thereby improving blood pressure control outcomes more effectively.

## 7 Future directions

Future research should focus on the following directions. First, studies should integrate objective data (e.g., ambulatory blood pressure monitoring, wearable device records) with qualitative interviews to verify the stability and explore the dynamic evolution patterns of the identified latent subtypes. Second, future studies should incorporate system-level variables (e.g., healthcare institution capacity, community environmental support) to develop a more comprehensive theoretical model of the factors influencing health behaviors. Third, introducing the chronic disease trajectory framework would allow researchers to systematically track transformation paths of patients’ health behavior subtypes across disease stages. Concurrently, exploring AI-based adaptive intervention strategies could provide a critical pathway toward precision health management.

## Data Availability

The original contributions presented in the study are included in the article/supplementary material, further inquiries can be directed to the corresponding author.
