# Correction: Latent profile analysis and influencing factors of proactive health behaviors in hypertensive patients from the perspective of the health belief model

**DOI:** 10.3389/fpubh.2026.1888852

**Published:** 2026-06-12

**Authors:** Zheyuan Xia, Yukuan Miao, Leran Tang, Ting Yao, Qiao Hu, Xiao Wang, Xiang Wang

**Affiliations:** 1 The School of Nursing, Anhui University of Chinese Medicine, Hefei, China; 2 Laboratory of Geriatric Nursing and Health, Anhui University of Chinese Medicine, Hefei, China; 3 Emergency Intensive Care Unit (EICU), The First Affiliated Hospital of Anhui Medical University, Hefei, China

**Keywords:** health belief model, hypertension, latent profile analysis, nursing care, proactive health

Reference 23 was erroneously written as “23. Narayanan H, Shankar K, Shankar SL. Cortisol levels and their association with workplace stress in IT workers. *Natl J Community Med*. (2025) 16:82430. doi: 10.1016/j.ijnurstu.2024.104808”. It should be “23. Narayanan H, Shankar K, Shankar SL, ShanmugaPriya K, Anbumaran PM, Krishnan R. Cortisol levels and their association with workplace stress in IT workers. *Natl J Community Med*. (2025) 16:824–30. doi: 10.55489/njcm.160820255534”.

Reference 26 was erroneously written as “26. Mentrup S, Harris E, Gomersall T. Patients‘ experiences of cardiovascular health education and risk communication: a qualitative synthesis. *Qual Health Res*. (2020) 30:88–104. doi: 10.1177/1049732319880551”. It should be “26. Mentrup S, Harris E, Gomersall T, Köpke S, Astin F. Patients' experiences of cardiovascular health education and risk communication: a qualitative synthesis. *Qual Health Res*. (2020) 30:88–104. doi: 10.1177/1049732319887949”.

The original version of this article has been updated.

